# The Clinical Non-Motor Connectome in Early Parkinson’s Disease

**DOI:** 10.3233/JPD-202102

**Published:** 2020-10-27

**Authors:** Nico J. Diederich, Nicolas Sauvageot, Vannina Pieri, Géraldine Hipp, Michel Vaillant

**Affiliations:** a Department of Neurology, Centre Hospitalier de Luxembourg, Luxembourg City, Luxembourg; b Competence Center for Methodology and Statistics, Luxembourg Institute of Health, Strassen, Luxembourg; c Luxembourg Centre of Systems Biomedicine, University of Luxembourg, University of Luxembourg, Belvaux, Luxembourg

**Keywords:** Parkinson’s disease, non-motor symptoms, connectome, compensation

## Abstract

**Background:**

Non-motor symptoms (NMS) of various anatomical origins are seen in early stage idiopathic Parkinson’s disease (IPD).

**Objective:**

To analyse when and how NMS are linked together at this stage of the disease.

**Methods:**

Prospective study recruiting 64 IPD patients with ≤3 years of disease duration and 71 age-matched healthy controls (HC). NMS were clustered in 7 non-motor domains (NMD): general cognition, executive function, visuospatial function, autonomic function, olfaction, mood, and sleep. Correlation coefficients ≥|0.3| were considered as significant. Bootstrapped correlation coefficients between the scores were generated in both groups. Fourteen IPD patients and 19 HC were available for a follow-up study two years later.

**Results:**

The mean age of both groups was similar. 58% of IPD patients and 37% of HC were male (*p* = 0.01). At baseline IPD patients performed less well than HC on all NMD (*p* value between 0.0001 and 0.02). Out of 91 possible correlations between NMD, 21 were significant in IPD patients and 14 in HC at the level of ≥|0.3|. The mean correlation level was higher in IPD patients than in HC, as evidenced by the higher box plot of correlation coefficients. Visuospatial scores at baseline were predictive of the motor deterioration at the follow-up exam.

**Conclusion:**

At early IPD stage various NMS are linked together, although not connected by anatomical networks. Such a clinical NMD connectome suggests almost synchronous disease initiation at different sites as also supported by fMRI findings. Alternatively, there may be compensation-driven interconnectivity of NMD.

## INTRODUCTION

Non-motor (NM) symptoms substantially contribute to the disease burden in idiopathic Parkinson's disease (IPD). In many patients they are forerunners of the core motor syndrome. In this perspective, the REM sleep behaviour disorder has been most thoroughly explored. Cognitive deficits, depression, hyposmia and other sleep disturbances have also been identified as forerunner symptoms [1, 2]. While early concomitant manifestation of various NM symptoms is inconsistent with Braak’s hypothesis of an ascending degeneration process starting in the lower brainstem and implying *sequential* manifestation of NM symptoms, it is consistent, however, with the concept of *synchronous* disease seeding by misfolded *α*-synuclein in different areas not limited to the basal ganglia and the brainstem, but including peripheral ganglia as well as the retina [3–5]. We hypothesized that, if there is such synchronous disease initiation in different areas, NM symptoms of different anatomical origins should be statistically linked to each other in early stage IPD. We address this hypothesis in the present exploratory study.

## METHODS

### Cohort recruitment

Non-demented IPD patients, as defined by the London brain bank criteria [6], were prospectively recruited at an early motor stage of the disease, defined as less than 3 years of disease duration. By mouth-to mouth propaganda 71 age-matched non-demented healthy controls (HC) were recruited as well.

### Methods

Tests and questionnaires with numerical scores were selected for the study, as they are appropriate for the statistical analysis described below. The assessment conditions had been described before [7]. In addition to the tests mentioned before [7], we also used the Behavioural Assessment of the Dysexecutive Syndrome (BADS). This test battery explores executive abilities related to frontal dysfunction. The subtests 2,5,6 entitled “Action Program Test”, “Zoo Map Test”, and “Modified Six Elements Test” were selected [8]. We further included the Visual Object and Space Perception Battery (VOSP) with the five subtests: screening, letters, object recognition, silhouettes, and gradual silhouettes [9]. The first four tests form the “VOSP total 1–4” score, which is a positive score The VOSP test exploring gradual silhouettes is considered separately, as it gives a negative score. Affirmative answers of the Non-motor questionnaire were summed up to a total score (NMS) [10]. At the beginning of the recruitment phase, we had used the questionnaire on the presence of REM sleep behaviour disorder (RBD), proposed by Comella et al. [11]. Later on we used the REM Sleep Behaviour Disorder Screening Questionnaire (RBDSQ). As both tests need a room partner for appropriate answering, their sensitivity in early stage of PD has been questioned [12, 13]. Because of these caveats and the inconsistency of the used questionnaires we only mention the results obtained on presence or absence of RBD as descriptive values. We clustered all the scores obtained into 7 non-motor domains (NMD): general cognition, executive function, visuospatial function, autonomic function, olfaction, mood, and sleep (Table 1).

**Table 1:** Seven non-motor domains and two UPDRS scales with their score ranges used in this study

Non -motor Domain	Test	Score Range
1. General cognition	•Mini-Mental State Examination (MMSE)	30–0
2. Executive function	•Frontal Assessment Battery (FAB)	18–0
	•Trail Making test A (TMTA)	(s) 0–∞
	•Behavioral Assessment of the Dysexecutive Syndrome (BADS)	12–0
3. Visuospatial function	•Color discrimination (Farnsworth-Munsell test)	0–400
	•Contrast sensitivity (Vis Tech)	45–0
	•Contrast sensitivity (Pelli-Robson)	2.25–0
	•Visual Object and Space Perception Battery (VOSP, subtests 1-4, total score)	90–0
	•VOSP gradual silhouettes	2–20
4. Mood	•Beck Depression Inventory (BDI)	39–0
5. Olfaction	•University of Pennsylvania Smell Identification Test (UPSIT)	40–0
6. Autonomic function	•Assessment of autonomic dysfunction (SCOPA-AUT)	0–69
	•Parkinson disease non-motor score (PDNMS)	0–24
7. Sleep	•Parkinson disease sleepiness score (PDSS)	0–150
	•REM sleep behavior disorder (RBD)	Yes/no
UPDRS III	•Motor score	0–56
UPDRS IV	•Activities of daily living	0–16

All the tasks were untimed. While performing the tests and questionnaires, the IPD patients and the HC subjects maintained their usual medications. The dosage of dopamine agonists was expressed in levodopa equivalence dosage (LEED) according to the formula: 1 mg pramipexole = 1 mg pergolide = 3 mg ropinirole = 10 mg bromocriptine [14]. The testing conditions were identical at the follow-up exam. Two experienced investigators provided all the tests (GH and VP). Before entering the study, all subjects had given informed written consent and the study had been approved by the “Comité National d'Éthique de la Recherche” (CNER N°200401/03) in Luxembourg.

### Statistical analysis

#### Scores at baseline exam T0

The results obtained in the neuropsychological tests were compared by the Kruskal-Wallis test. Correlation coefficients between the scores were generated separately in IPD patients and HC by the non parametric Spearman test. In order to obtain confidence intervals and more reliable results, bootstrapped correlation coefficients were computed [15, 16]. Thus 1000 simple random samples with replacement of observations, each of the same size, were randomly drawn for the IPD patients and the HC. Bootstrap correlation coefficients were first computed, followed by averaged correlation coefficients with 95% confidence intervals. In order to establish a table of the correlation coefficients between NM tests and questionnaires of different NMD, we considered only the correlation coefficients higher or equal to 0.3. Correlation coefficients within the same NMD are reported separately. The correlation coefficients between NM scores and UPDRS motor or ADL scores were discarded, therefore totalling 91 possible correlations. By the Spearman correlation we calculated the predictive value of different NM scores obtained at the exam time T0 on motor deterioration, defined as score of the UPDRS motor at T1 minus score of the UPDRS motor score at T0.

#### Comparison of the scores between T0 and T1

In IPD patients and controls with both T0 and T1 values Spearman rank correlations were used in order to calculate the change of the scores between both testing dates. The modelling was carried out through a shrinkage method of the variable selections. Variables were first selected using the elastic net procedure, which mixes the so called “least absolute shrinkage and selection operator” (LASSO) procedure and ridge regression [17]. In a second step model averaging was achieved by using the above described bootstrap methodology. Finally, we applied a linear model for the changes of the UPDRS motor and ADL scores between the T0 and T1 evaluations. Of note, in the covariance analysis the scores at T0 were included in the explaining variables, concomitantly to the subject groups IPD or HC, and the changes in the different tests. We used a generalized linear model (GLM) after Elasticnet variable selection.

## RESULTS

### Demographics

We recruited 64 IPD patients and 71 healthy controls for the baseline exam. Both groups did not differ in terms of age (IPD patients 64.2±11.6 years, and HC 64.7±8.6 years). There was no difference concerning the duration of education (IPD patients 12.6±3.6 years and HC 13.1±3.5 years). There was a mild preponderance of male IPD patients (58% versus 37% of HC; *p* = 0.01). IPD patients were on a levodopa dosage of 177.4±276.2 mg and a LEDD of 0.7±1.6 mg per day.

### Baseline exam

**Fig. 1:**
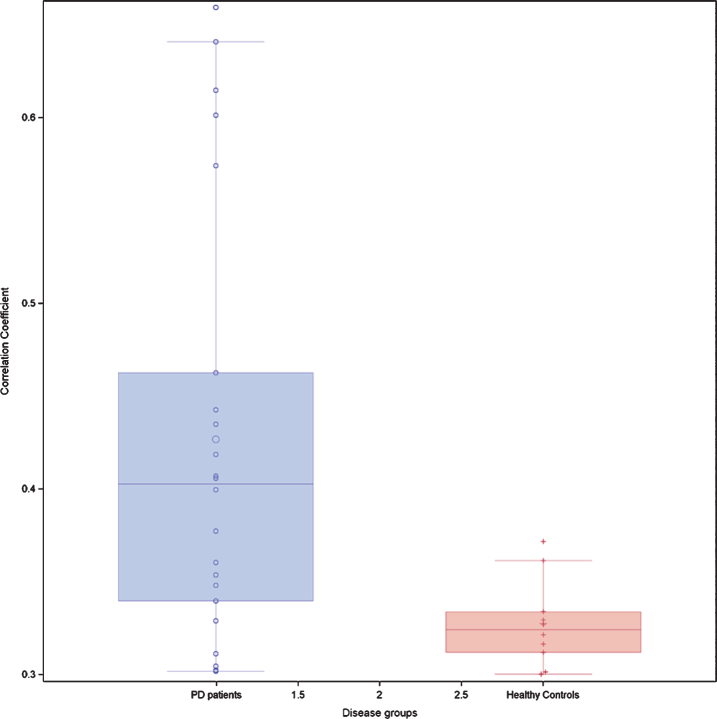
Box-plots of all possible correlation coefficients (*n* = 91) between the seven non-motor domains in 64 patients with idiopathic Parkinson’s disease and 71 healthy controls at the baseline exam T0. Each circle represents an individual correlation coefficient.

**Fig. 2:**
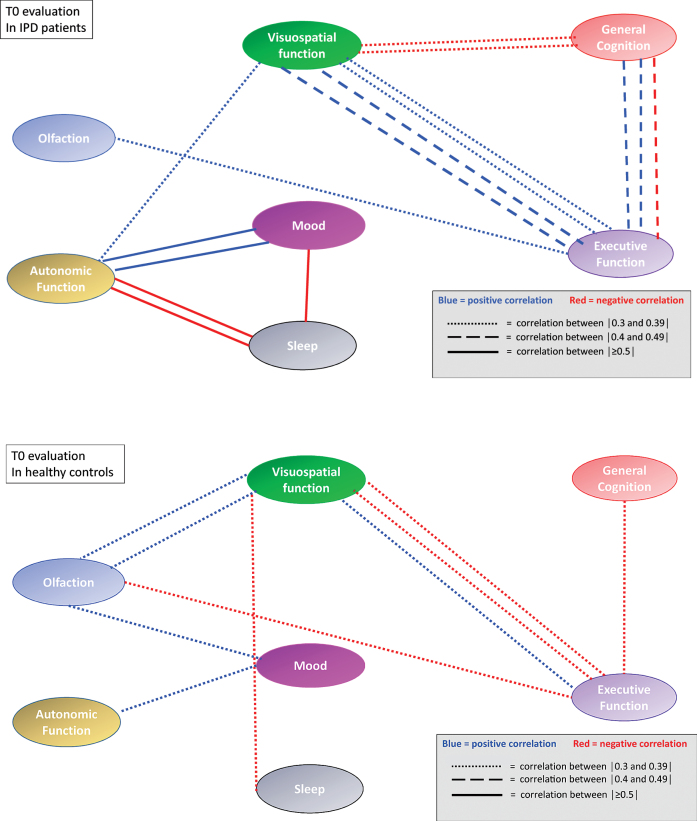
Correlation network (“connectome”) based on correlation coefficients between different non-motor domain scores at T0 in IPD patients and HC. The strength of the correlation is indicated by different dashes of the connecting lines. Negative correlations are indicated by red lines, positive correlations by blue lines. Correlations between different variables within a non-motor domain are not represented.

At baseline IPD patients performed less well than HC on all NMD (p between <0.0001 and 0.02) (Table 2). Seven (10.9%) IPD patients and two (2.8%) HC had a positive RBD score. When applying the rules on correlation coefficients as established above, there were in total 21 significant correlation coefficients at T0 between the different NM scores in IPD patients and 14 in HC. When considering only the correlation coefficients between *different* NMD, there were 16 significant correlations coefficients in IPD patients and 10 in HC. In IPD patients, 3 correlations were each time seen between the score of the SCOPA-AUT questionnaire, respectively the score of the Farnsworth test and other NM domains. In IPD patients, three out of five significant correlation coefficients *within the same* NMD concerned the visuospatial function, while in HC subjects only one of four significant correlation coefficients within the same NMD concerned the visuospatial function. The correlation links were not identical in IPD patients and HC (Table 3A, B). When considering all possible correlations between NM scores, the mean correlation level (absolute value) was 0.40 in IPD patients and 0.33 in HC. This was supported by the box plot of the correlation coefficients where the 25th percentile, the median and the 75th percentile were higher in IPD than in HC (Fig. 1). The correlation network (“connectome”) based on the different correlation coefficients is presented in Fig. 2, which also indicates the strength of the correlation as well as the positive or negative type of correlation.

**Table 2:** Performances of healthy controls and patients with idiopathic Parkinson’s disease at baseline examination T0. The results are classified in 7 non-motor domains

	Idiopathic Parkinson disease patients			Healthy controls			Effect Size	Kruskal-Wallis
Test	N	mean	SD	n	mean	SD		*p*-value
General cognition
MMSE	64	28.80	1.40	71	29.37	0.90	-0.57	0.02
Executive function
FAB	64	15.88	1.68	71	17.03	1.18	–1.15	<0.0001
TMT A (time s)	64	47.77	19.70	71	39.35	13.30	8.41	0.018
BADS	58	5.71	1.61	67	6.09	1.40	–0.38	0.17
Visuospatial function
Farnsworth	64	121.14	71.52	71	88.45	59.00	32.69	0.007
Vistec	64	20.23	5.92	71	22.80	5.13	–2.57	0.01
Pelli-Robson	53	1.45	0.24	60	1.61	0.21	–0.16	0.0001
VOSP total 1–4	58	53.26	8.28	67	55.64	5.55	–2.38	0.16
VOSP gradual silhouettes	58	10.67	3.08	67	10.67	2.67	0.00	0.87
Mood
Beck Depression Inventory	62	6.15	5.36	67	3.43	2.71	2.71	0.0003
Olfaction
UPSIT	64	20.73	8.04	71	29.07	5.57	–8.34	<0.0001
Autonomic functions
Scopa AUT	58	14.95	10.54	67	8.69	6.26	6.26	0.0002
PDNMS	42	7.95	5.41	66	4.77	3.35	3.18	0.0005
Sleep
PDSS	58	94.71	37.21	67	108.33	36.31	–13.62	0.003
REM behavior disorder	64	7.00	10.90%	71	2.00	2.80%	8.10%	0.08^*^
UPDRS scores
UPDRS III (motor)	64	9.23	5.18	71	0.51	1.19	8.73	<0.0001
UPDRS IV (ADL)	58	5.21	4.31	67	0.30	0.94	4.91	<0.0001

SD, standard deviation; ^*^Fisher *p*-value.

**Table 3:** 3A and B. Correlation boxes between different non-motor scores obtained at base line exam T0 by patients with idiopathic Parkinson’s disease patients (IPD) and healthy controls (HC). When the absolute value ≥than 0.3 and the scores relate to different non-motor domains, the correlation coefficient is marked in yellow. When the scores relate to tests within the same non-motor domain, the correlation coefficient is marked in green

A
IPD patients at T0	FAB	TMTA	BADS	Farnsworth	Vistec	VOSP gradual silhouettes	Pelli Robson	BDI	UPSIT	Scopa AUT	PDNMS	PDSS	VOSP total
MMSE	0.44	–0.41	0.43	–0.30	0.08	–0.30	0.07	–0.28	0.13	–0.21	–0.22	0.19	0.24
FAB		–0.08	0.28	0.15	0.19	–0.29	0.11	–0.28	0.30	–0.19	–0.27	0.13	0.33
TMTA			–0.51	0.36	–0.25	0.41	–0.20	0.14	–0.05	0.13	0.07	–0.07	–0.24
BADS				–0.26	0.22	–0.27	0.14	–0.13	0.22	–0.09	–0.04	0.09	0.42
Farnsworth					–0.38	0.06	–0.30	0.11	–0.06	0.35	0.17	–0.24	0.02
Vistec						–0.15	0.76	–0.07	0.27	–0.28	–0.08	–0.01	–0.03
VOSP gradual silhouettes							–0.11	0.01	–0.16	–0.14	0.01	0.20	–0.25
Pelli Robson								–0.05	0.25	–0.25	–0.13	0.04	–0.11
BDI									0.06	0.57	0.66	–0.61	0.14
UPSIT										–0.05	0.14	–0.10	0.01
Scopa AUT											0.58	–0.64	0.11
PDNMS												–0.60	0.05
PDSS													–0.10
B
HC at T0	FAB	TMTA	BADS	Farnsworth	Vistec	VOSP gradual silhouettes	Pelli Robson	BDI	UPSIT	Scopa total	PDNMS	PDSS	VOSP total
MMSE	0.04	–0.33	0.19	0.10	0.15	–0.24	0.25	0.03	0.13	–0.08	–0.01	0.07	0.03
FAB		–0.36	0.37	0.02	0.11	–0.32	0.00	0.10	0.24	–0.19	–0.08	0.15	–0.05
TMTA			–0.13	0.13	–0.31	0.37	–0.28	–0.16	–0.33	0.08	–0.02	–0.24	–0.06
BADS				–0.04	0.17	–0.04	0.08	–0.08	0.03	–0.17	–0.02	0.15	0.04
Farnsworth					–0.19	0.03	–0.18	0.05	–0.06	–0.16	–0.04	–0.01	0.18
Vistec						–0.01	0.62	0.08	0.32	–0.07	–0.14	0.19	–0.02
VOSP gradual silhouettes							–0.08	–0.13	–0.06	–0.05	–0.12	–0.10	0.18
Pelli Robson								0.15	0.33	0.06	–0.08	0.28	–0.14
BDI									0.36	0.20	0.30	–0.02	0.11
UPSIT										0.10	0.01	–0.06	0.22
Scopa AUT											0.42	–0.09	0.02
PDNMS												–0.16	0.09
PDSS													–0.30

### Follow-up evaluation

Due to logistic and financial recruitment limitations, only 14 patients and 19 healthy controls were available for a follow-up exam T1 after a median duration of 2.03 years in IPD patients and 2.30 years in HC. However, these subjects were similar to the whole group in terms of age and gender distribution. The results obtained are reported in the Supplementary Table 1.

### Changes of the NM scores between baseline and follow-up exam

We calculated the correlation between the changes of the scores obtained by the subjects at the baseline exam T0 and the follow-up exam T1. IPD patients showed 18 significant correlation coefficients above 0.3 between score changes in different NMD and 3 additional significant correlation coefficients between score changes within the same NMD domain. The latter concerned twice the visuospatial domain and once the executive domain. In contrast, in HC subjects we found only 11 significant correlation coefficients above 0.3 between different NMD and two additional significant correlation coefficients within the visuospatial domain (Table 4A, B).

**Table 4:** 4A and B. Correlation boxes between the differences of the non-motor scores obtained by patients with in idiopathic Parkinson disease patients (IPD) and healthy controls (HC) at T1. When the absolute value is ≥than 0.3 and the scores relate to different non-motor domains, the correlation coefficient is marked in yellow. When the scores relate to tests within the same non-motor domain, the correlation coefficient is marked in green

A
Difference between T1 and T0 in IPD	FAB	TMTA	BADS	Farnsworth	VISTEC	VOSP gradual silhouettes	Pelli Robson	BDI	UPSIT	Scopa total	PDNMS	PDSS	VOSP total
MMSE	0.05	–0.07	0.28	–0.61	–0.13	–0.11	0.11	–0.39	–0.07	–0.03	0.09	0.08	0.09
FAB		0.64	–0.05	0.32	0.26	–0.40	0.00	–0.34	0.03	–0.39	–0.24	0.07	–0.42
TMTA			0.12	–0.01	–0.04	0.07	–0.14	0.23	0.26	–0.55	–0.02	0.06	0.10
BADS				–0.26	0.09	–0.37	–0.15	0.41	0.43	–0.39	0.17	0.34	–0.07
Farnsworth					0.16	–0.13	–0.21	–0.06	–0.23	–0.02	–0.31	0.06	–0.55
VISTEC						–0.12	0.71	–0.07	0.31	0.37	–0.01	0.27	–0.15
VOSP gradual silhouettes							0.11	0.06	–0.09	0.34	0.34	0.24	0.53
Pelli-Robson								–0.13	0.02	0.24	0.00	0.13	0.24
BDI									0.39	–0.20	0.19	0.17	0.33
UPSIT										0.00	–0.20	0.08	–0.07
Scopa-Aut											0.07	0.01	–0.02
PDNMS												0.54	0.57
PDSS													–0.05
B
Controls	FAB	TMTA	BADS	Farnsworth	VISTEC	VOSP gradual silhouettes	Pelli Robson	BDI	UPSIT	Scopa Aut	PDNMS	PDSS	VOSP total
MMSE	0.10	–0.20	0.32	–0.24	–0.27	0.17	0.02	–0.16	–0.35	–0.42	0.35	–0.14	–0.22
FAB		0.46	0.11	0.22	0.01	0.23	0.36	0.12	–0.01	0.10	0.01	–0.15	0.25
TMTA			–0.32	0.16	0.07	0.19	0.08	0.18	–0.09	0.30	0.00	–0.10	0.11
BADS				0.13	–0.05	0.29	0.03	–0.29	–0.18	–0.54	–0.24	0.11	0.13
Farnsworth					–0.13	–0.08	–0.08	0.27	0.36	0.14	–0.24	–0.19	0.27
VISTEC						0.18	0.12	–0.05	0.02	0.16	–0.40	–0.29	0.10
VOSP gradual silhouettes							–0.02	–0.42	–0.37	0.23	–0.22	0.00	0.15
Pelli-Robson								0.27	–0.04	–0.16	0.09	0.25	–0.13
BDI									0.49	–0.05	–0.06	–0.09	0.06
UPSIT										–0.14	0.14	–0.04	0.11
Scopa-Aut											–0.26	–0.26	0.23
PDNMS												0.08	–0.64
PDSS													–0.17

### Predictive value of NM scores at T0 on motor deterioration

The selected variables (above 7% of selection in the model averaging process) were “group” and “VISTEC” for both the UPDRS motor and the UPDRS ADL scores (data not shown). PDNMS was later discarded because it was not significant in the model. The baseline evaluation of each score and the interaction with the patient group were added to each model in order to evaluate the difference between groups. The changes in the UPDRS motor and ADL were estimated from the models by re-calculating them from the beta estimates of the models. The significant estimates are presented in Table 5.

**Table 5:** Prediction of the change in motor degradation between the baseline T0 and follow-up T1 exams by PD status (IPD or Healthy) and VISTEC score T0–T1 change. The intercept indicates the mean basic change in motor degradation (GLM model after Elasticnet variable selection)

Parameter	Estimate	Standard Error	*t* Value	Pr > | t|	Change in Motor score
Intercept	0.25	0.57	0.45	0.66
Motor impairment at T0	–0.40	0.11	–3.53	0.002
IPD patients	7.30	1.33	5.49	<0.0001	+6.12
Healthy controls	0				+0.13
Change of Vistec score	0.28	0.20	1.4	0.17
Change of Vistec score in IPD patients	–1.32	0.29	–4.53	<0.0001
Change of Vistech score in healthy controls	0

The UPDRS motor score at T1 was negatively influenced by the VISTEC at T0. Higher baseline scores in VISTEC predicted less pronounced deterioration of the UPDRS motor score between T0 and T1. In IPD patients, but not in HC, the change of the ADL score between T0 and T1 in IPD patients was similarly predictable by the baseline VISTEC score (data not shown).

## DISCUSSION

NM symptoms are widely present at the early IPD stage and contribute substantially to the quality of life of the patients [18]. They have different pathophysiological causes and they implicate different sites or networks. At first look, there are no functional nor anatomical links between them [19, 20]. However, in the present study there has been at baseline a significantly denser correlation “network” between disparate NM symptoms in IPD patients than in HC. The results suggest the existence of a *clinical* NM network or connectome with specific NMD even acting as nodes or hubs. Why is such a network seen in IPD patients, but not so in HC subjects? The answer could be multilayered, and we propose several possible explanations.

First, some proposed NMD represent overlapping or highly interdependent functions. In particular, complex NM functions such as sleep or mood are executed by several anatomical locations and depend on the interplay of several neuro-amine systems. Therefore, if these NMD become deficient, they may also correlate with each other, as seen between different scores exploring depression and sleep, or between different scores of the PDNMS scale. However, the present study shows correlations beyond such self-evident correlations, for instance between color discrimination (Farnworth) and autonomic dysfunction (Scopa-AUT). In total, there have been seven significant correlation links between visuospatial scores and other NMD scores. Consequently, explaining the correlation network by just overlapping functions or intrinsic interdependency has to be refuted.

Second, there may be just parallel, although unrelated, disease progression in different non-motor domains. Parallelism of progression could be due to synchronous disease initiation at different sites, as formulated in our initial hypothesis. It is also possible that a hidden third - not yet measurable - factor to which two NMD A and B would be linked, produces pseudo-correlations between A and B. Increased or decreased attention or vigilance could be such a factor. However, such a hidden factor should, similarly impact the performances in IPD patients and HC, which is not the case.

Third, could the visuospatial performances alone explain (almost) all results? Indeed, in this study visuospatial performances were highly linked to scores in four other NMD, and the score obtained in one visual contrast exam at baseline (VISTEC) predicted the motor deterioration seen in IPD patients at the follow-up exam. The results corroborate earlier findings showing that visual deficits alone can discriminate early IPD patients from HC [7]. However, these findings cannot explain other constituents of the presumed connectome, not linked to the visuospatial domain.

Fourth, beyond the data of this study, functional magnetic resonance imaging (fMRI) studies may give a more comprehensive explanation for all our findings. Indeed, analysis of the temporal fluctuations of functional connectivity has shown that IPD patients more frequently than HC are at resting state in a widely interconnected “between network” state rather than a “within network” state [21, 22]. In other words, there is frequently a functional connectivity state, spontaneously linking disparate networks such as visual, cognitive, executive or the default mode network (DMN). In particular, increased functional connectivity with the visual network has been reported [21, 23], confirming our findings of several correlation links between visuospatial performances and other NMD, as well as the predictive potential of visual deficits on motor deterioration. In IPD patients with mild cognitive impairment including executive dysfunction, the DMN also shows increased connectivity with occipito-parietal regions [24]. All these neuroimaging findings suggest wide-ranging network dysfunction. However, such results are different from histopathological proof of disease involvement at different neuroanatomical sites. Having said that, fMRI data are nevertheless strongly supportive of our concept of a clinical connectome between various NMD in IPD patients. More generally, these changes, both in terms of interrelation of clinical signs and of functional connectivity, confirm the statement by Tinaz et al. [25] that in IPD non-motor signs have to be “approached as a phenomenon emerging from the abnormal connections and interactions between different brain regions rather than being the result of focal lesions.”

Finally, and as alternative explanation, compensatory mechanisms at an early IPD stage may explain, both increased clinical and fMRI interconnectivity. Being a general principle of ongoing neurodegeneration [26], it may already be present at a preclinical stage, as suggested by fMRI findings of successful compensation in subclinical carriers of genetic Parkinsonian syndromes [27–29]. Presumably, compensatory mechanisms have only temporarily limited efficiency; nevertheless, in depth investigation of these mechanisms seems promising, as they could reflect natural defence mechanisms against IPD progression. Due to the reduced number of subjects recruited for the follow-up exam, our dataset has not been robust enough to confirm or refute the hypothesis that, with progressing disease however, the correlation network becomes again looser because synaptic losses now produce a disconnection syndrome or connectome dissolution [30].

The strengths of our study include the extensive evaluation of sensory deficits including colour discrimination, contrast sensitivity, visuospatial performances, all rarely examined in such detail in IPD patients. The use of bootstrap correlation coefficients is also an innovative statistical approach. However, this has been an exploratory study with a limited number of participants, in particular at the follow up exam T1. A selection bias seems to be excluded, as the demographics of the cohorts at T0 and T1 are statistically comparable. Nevertheless, confirmation in larger cohorts of the data obtained is warranted. Of note, we do not consider our study to be biased by the exclusion of categorical scores, as the obtained numerical scores adequately cover all non-motor domains.

In conclusion and by coming back to the initial hypothesis, we found a correlation network between various clinical non-motor symptoms in IPD patients at an early stage of the disease, thus establishing a clinical connectome in these patients. These findings, while being consistent with synchronous disease initiation at different sites, can also be explained by other mechanisms as described above. The “widespread branching” confirms recent fMRI data showing similar widespread interconnectivity. It may also be compatible with the concept of transiently efficient compensatory mechanisms in neurodegeneration. We suggest intensified research efforts focusing on a better understanding of such interrelationships between sensory and other NM symptoms at an early stage of IPD as well as exploring early compensation mechanisms. Both research avenues may detect yet unknown opportunities for therapeutic applicability.

## CONFLICT OF INTEREST

The authors have no conflict of interest to report.

### Data availability

The complete dataset of this study is available on request by the senior author MV (michel.vaillant@lih.lu).

## Supplementary Material

Supplementary Material
