# Supplementary material for: The Clinical Non-Motor Connectome in Early Parkinson’s Disease
Source: J Parkinsons Dis. 2020 Oct 27;10(4):1797–806. doi: 10.3233/JPD-202102 (PMC7683075; doi:10.3233/JPD-202102)
Supplement: Supplementary Material [file jpd-10-jpd202102-s001.pdf]

Supplementary Material

The Clinical Non-Motor Connectome in Early Parkinson's Disease

Supplementary Table 1. Performances of healthy controls and patients with idiopathic Parkinson's disease (IPD) at follow-up examination T1. The results are classified in 7 non-motor domains.

	IPD patients			Healthy controls			Effect Size	Kruskal-Wallis
**Test**	**n**	**mean**	**SD**	**n**	**mean**	**SD**		**p-value**
**General cognition**								
**MMSE**	14	28.9	1.1	19	29.5	0.8	-0.6	0.06
**Executive function**								
**FAB**	14	15.1	1.6	19	16.3	1.5	-1.2	0.04
**TMT A (time s)**	14	53.9	29.3	19	40.3	10.7	13.5	0.10
**BADS**	14	5.4	1.3	19	6.2	1.6	-0.9	0.09
**Visuospatial function**								
**Farnsworth**	14	117.4	43.4	19	69.8	45.8	47.6	0.009
**Vistec**	14	18.6	5.2	19	22.3	6.1	-3.7	0.05
**Pelli-Robson**	14	1.4	0.2	19	1.5	0.2	-0.09	0.11
**VOSP total 1-4**	14	51.6	9.6	19	56.0	5.2	-4.4	0.13
**VOSP gradual silhouettes**	14	10.2	2.8	19	9.5	2.9	0.7	0.44
**Mood**								
**Beck Depression Inventory**	14	7.4	6.3	19	3.2	2.0	4.2	0.05
**Olfaction**								
**UPSIT**	14	18.6	4.9	19	28.0	6.4	-9.4	0.0003
**Autonomic functions**								
**Scopa AUT**	14	19.6	8.9	19	8.5	5.4	11.1	0.0006
**PDNMS**	14	9.8	5.2	19	4.7	2.9	5.1	0.003
**Sleep**								
**PDSS**	14	101.3	28.8	19	109.9	27.2	-8.7	0.48
**REM behavior disorder**	14	3	21.4%	19	1	5.3%	16.2%	0.2*
**UPDRS scores**								
**UPDRS III (motor)**	14	13.5	5.9	19	0.5	2.3	13.0	<0.0001
**UPDRS IV (ADL)**	14	8.3	6.2	19	0.1	0.3	8.2	<0.0001

SD, standard deviation; *Fisher p-value
